# Drug-induced interstitial lung disease due to over-the-counter cold medicine taken daily for 25 years: a case report

**DOI:** 10.1186/s13256-022-03717-9

**Published:** 2023-02-27

**Authors:** Masataka Matsumoto, Isao Ito, Seizo Kadowaki

**Affiliations:** 1 Department of Respiratory Medicine, Kitaharima Medical Center, 926-250 Ichiba-Cho, Ono, Hyogo 675-1392 Japan; 2 Department of Respiratory Medicine, Kyoto University Hospital, 54 Shogoin-Kawaharacho, Sakyo, Kyoto, 606-8507 Japan; 3 Department of Internal Medicine, Ono Municipal Hospital, 323 Naka-Cho, Ono, Hyogo 675-1332 Japan

**Keywords:** Drug-induced interstitial lung disease, Over-the-counter, Acetaminophen, Cold medicine, Side effect

## Abstract

**Introduction:**

We report a rare case of drug-induced interstitial lung disease due to over-the-counter cold medicine taken daily for 25 years to clear the patient’s head.

**Case presentation:**

A 77-year-old Japanese man presented to our hospital with a worsening cough that started 5 years ago. Chest radiographs and computed tomography images showed bilateral opacities, and transbronchial lung biopsy specimens showed an organizing pneumonia pattern. He reported taking the same over-the-counter cold medicine daily for the past 25 years to clear his head. We suspected that the cold medicine caused the lung opacities and asked him to stop taking them. His cough, general fatigue, and chest infiltrate gradually diminished. However, 6 months later, he resumed the same treatment because of a cold. The following month, he presented with severe worsening cough and chest radiographical findings. We diagnosed drug-induced interstitial lung disease. He improved by stopping the cold medicine again and taking prednisolone.

**Conclusions:**

Over-the-counter cold medicines are easily accessible at the drugstore. In cases of diffuse lung disease, we should consider drug-induced interstitial lung disease due to over-the-counter cold medicine, which patients have been taking not only for weeks or months but also years.

## Introduction

When we catch a cold, we can get an over-the-counter (OTC) cold medicine from any drugstore. The pharmaceutical affairs law in Japan does not require drugstore registered sellers to always ask in detail about symptoms, and they are not obligated to explain the effects and side effects of the medicine. If more cold medicine is required on another occasion or in another store, the drugstore clerk might sell it without checking the patient’s medication history. Although the prevalence is low, drug-induced interstitial lung disease cases due to OTC cold medicine are being reported [1]. The mechanisms through which drug-induced lung disease occurs are likely to involve direct damage to alveolar epithelial or capillary endothelial cells, dysregulation of the immune system, systemic cytokine release, cell-mediated lung damage, and free radical production with oxidative injury [2]. The drug-induced lymphocyte stimulation test (DLST) is not always positive [3]. Some OTC cold medicines contain codeine, dihydrocodeine, and caffeine; some patients might continue taking the medicine due to the addictive effects. However, drug-induced interstitial lung disease after long-term use has never been reported. We report an extremely rare case of drug-induced interstitial lung disease due to OTC cold medicine taken every day for 25 years.

## Case presentation

In July 2011, a 77-year-old Japanese man presented to our hospital with a worsening dry cough that started 5 years ago. He had a history of myocardial infarction (11 years ago) and prostate cancer (6 years ago). He had a smoking history of 23 pack-years from 20 to 43 years of age, no alcohol abuse history, no known food or drug allergies, and no history of home/occupational dust exposure (mold, down, bird droppings, or isocyanate). After myocardial infarction, he had been treated with aspirin, losartan potassium, famotidine, and simvastatin for 11 years. In addition, he was given goserelin, bicalutamide, or leuprorelin for prostate cancer for 15 years.

**Fig. 1:**
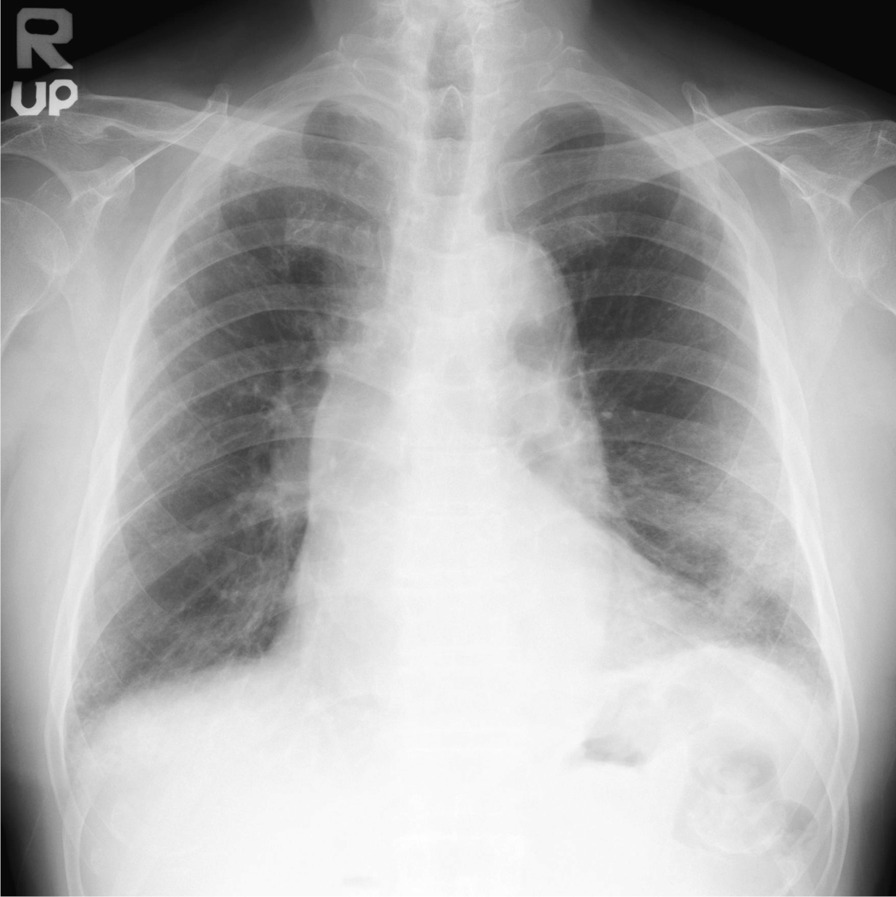
Chest X-ray at the first visit

**Fig. 2:**
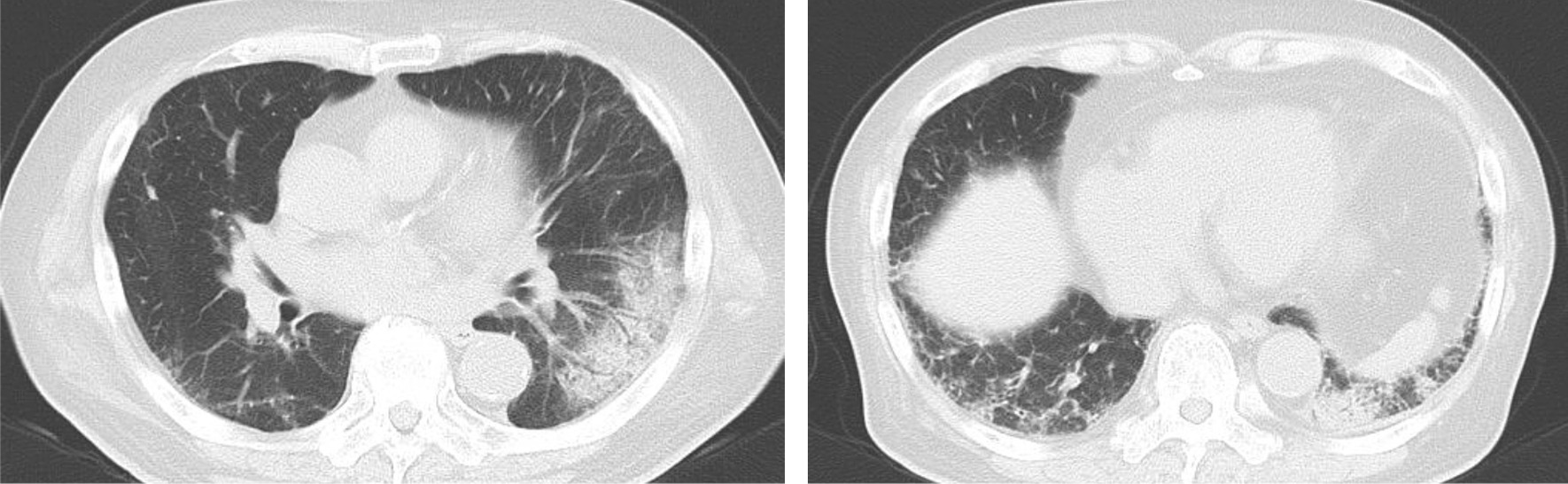
Chest CT at the first visit

**Fig. 3:**
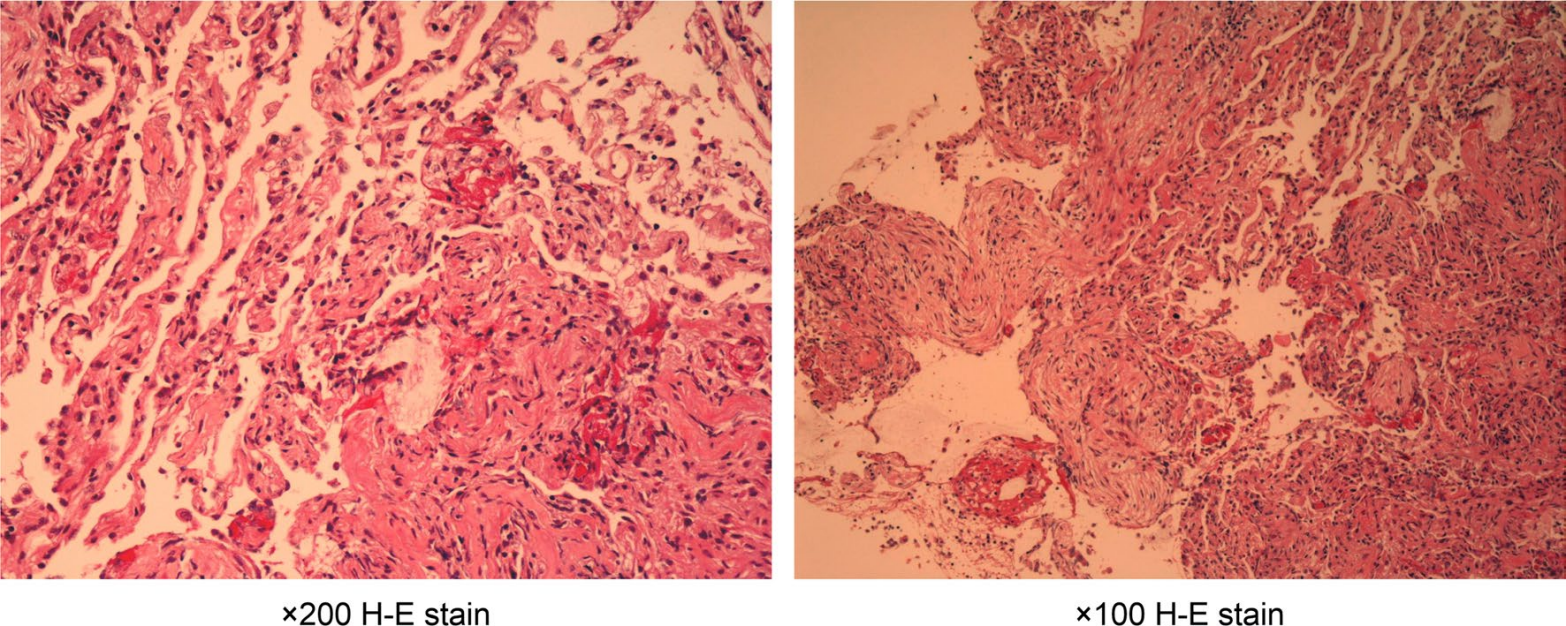
Transbronchial lung biopsy tissue from left B9a

The patient’s body mass index (24.3 kg/m^2^; weight 67 kg, height 1.66 m), body temperature (36.8 °C), blood pressure (161/91 mmHg), pulse rate 86 beats/minute), and respiratory rate (18 breaths/minute) were measured. Auscultation revealed bilateral fine crackles in both lower fields. No other abnormalities were observed. Laboratory tests showed mild anemia (hemoglobin 11.4 g/dL), elevated C-reactive protein (12.37 mg/dL), and slightly elevated KL-6 (562 U/ml). Arterial blood gas analysis on room air showed a low oxygen pressure of 68.3 mmHg. Chest radiograph and computed tomography (CT) images showed ground-glass opacity in the left lower lobe and reticular opacities in both bottom lobes (Figs. 1 and 2). Pulmonary function tests showed a restrictive respiratory pattern with a reduced vital capacity of 76.2% predicted and decreased diffusion capacity for carbon monoxide (alveolar ventilation of 63.1% predicted). Bronchoscopy revealed that bronchoalveolar lavage fluid (BALF) showed cell differentials of 70% macrophages, 22% lymphocytes, and 8% neutrophils. The CD4-to-CD8 lymphocyte ratio was 0.48. Pathogenic bacteria were not isolated from the sputum or BALF. Fibroblast proliferation was widely observed in the alveoli of the biopsy specimens taken from the left B9a (Fig. 3). We diagnosed organizing pneumonia based on the pathological findings.

A detailed interview revealed that to prevent a common cold and to clear his head, the patient had taken an OTC cold medicine (New lulu-A Tablet, Daiichi Sankyo Healthcare Co., Ltd.) daily for the past 25 years. The components of the cold medicine included acetaminophen, clemastine fumarate, noscapine, potassium guaiacol sulfonate, lysozyme hydrochloride, dihydrocodeine phosphate, dl-methylephedrine hydrochloride, anhydrous caffeine, and benfotiamine (vitamin B1 derivatives).

We asked the patient to stop taking the cold medicine while continuing other medications as prescribed. His cough, general fatigue, and chest infiltrates gradually diminished. DLST of the cold medicine was negative (148 cpm, Stimulation Index (SI) 135%).

**Fig. 4:**
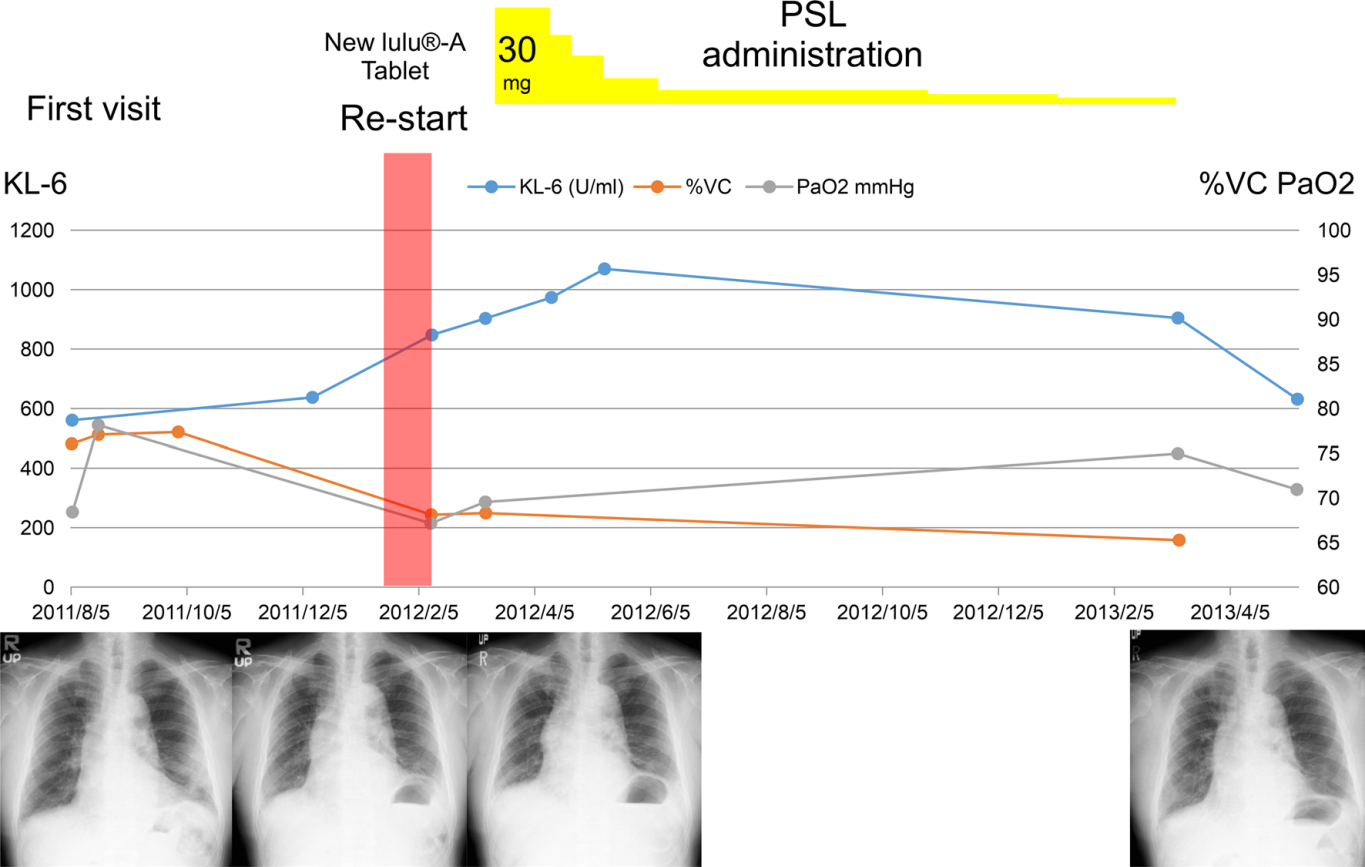
Patient’s course

The patient caught a cold on 5 January 2012, and again started taking the cold medicine daily. On 10 February, he presented to our hospital with a severe worsening of cough. Based on chest radiograph, spirometry, and arterial blood gas data (Fig. 4), we recommended discontinuation of the medicine again. Due to the recurrence of the lung disease after restarting the medicine, the patient was diagnosed with drug-induced interstitial lung disease due to the cold medicine. We considered that it was different from hypersensitivity pneumonitis because of the patient’s environment. From 9 March 2012, we administered 30 mg oral prednisolone daily (Fig. 4).

His cough, sputum, and dyspnoea gradually reduced, and radiographical and arterial blood oxygenation findings improved (Fig. 4). DLST for the cold medicine ingredients (acetaminophen, clemastine fumarate, noscapine, and potassium guaiacol sulfonate) were within normal limits. The patient was healthy at the time of this writing, with almost no respiratory system symptoms.

## Discussions

We encountered a man patient with drug-induced interstitial lung disease due to administration of the same OTC cold medicine daily for 25 years.

Many drugs have been associated with pulmonary complications of various types, including interstitial inflammation and fibrosis, bronchospasm, pulmonary edema, and pleural effusions [4]. Drug-induced interstitial lung disease is defined as a drug-related disorder in the respiratory system that occurs during administration of drugs including all OTC medicines, herbal medicines, supplements, narcotics, and drugs (for example, cancer drugs, rheumatology drugs, amiodarone, and antibiotics) prescribed by doctors [2, 5, 6]. Risk factors for drug-induced interstitial lung disease include old age (60 years or older), existing lung lesions, lung operation, low respiratory function, ongoing oxygen therapy, radiation for lung, multiple antimalignancy drug administration, and kidney dysfunction [5]. The proportion of drug-induced interstitial lung disease is 0.01–0.0025% in clinical trials in Japan, with the proportion increasing in the twenty-first century [5, 7].

The diagnostic criteria for drug-induced interstitial lung disease are as follows: (1) a history of administration of the causative drug; (2) clinical, radiological, or pathological findings consistent with previous reports on the suspected drug; (3) exclusion of causes except for drugs; (4) improvement after stopping the suspected drug; and (5) symptoms returning after readministration of the suspected drug [8]. Since DLST is reported to be positive only in 66.9% of cases, it is not essential for diagnosis [3].

The patient was given many drugs (aspirin, losartan potassium, famotidine, simbastatin, goserelin, bicalutamide, leuprorelin) that could cause drug-induced interstitial lung disease. However the observation that pneumonia worsened after restarting OTC cold medicine led us to conclude that the medicine had caused pneumonia.

From April 2004 to November 2016, side effects of OTC cold medicine in Japan were reported in 1276 cases, including 88 cases involving the respiratory system, thorax, and mediastinum (fourth place) [1]. Besides acetaminophen, drug-induced interstitial lung disease caused by ibuprofen-containing OTC cold medicine has been reported. Particularly for the drug in the present case, nine interstitial lung disease cases, including one death, were reported from 2007 to 2015. During direct communication, the company reported that 3.4 million packs of this OTC medicine are released annually [9].

The causative component of the OTC cold medicine was unknown in the case presented here. This drug contains acetaminophen, and more than 20 cases of acetaminophen-induced drug-induced interstitial lung disease have been reported [10–22]. Drug-induced interstitial lung disease due to any other component of this drug has not been reported. Thus, we assume acetaminophen to be the probable cause of interstitial lung disease in our patient. However, we are not sure that other components do not cause drug-induced interstitial lung disease. We will look for reports on drug-induced interstitial lung disease due to the other components in future.

Pathological findings in the present case showed an organizing pneumonia pattern. Drug-induced interstitial lung disease caused by acetaminophen commonly presents an eosinophilic pneumonia pattern [10–12, 15, 21, 22]. The organizing pneumonia pattern is usually seen in cases with a subacute clinical presentation. Drug-induced interstitial lung disease often develops over 2–3 weeks to 2–3 months [5]. We are not sure when our patient’s lung disease developed during the 25-year course of his medication history. Our observation that his vital capacity did not recover to normal suggests that some chronic irreversible pathological changes had also developed over the long course of the disease.

In general, the addictive effects of OTC drugs are low. However, a survey on psychiatric disorders due to drugs in Japanese registered psychiatric facilities from September 2018 to October 2018 found that addictive drugs used within 1 year are (1) stimulants (452 cases: 39.3%), (2) hypnotics/antidepressants (343 cases: 17.1%), and (3) OTC medications (105 cases: 9.1%, of which 83.3% were OTC cold medicine) [23]. In particular, some OTC cold medicines contain addictive codeine, dihydrocodeine, and caffeine.

In many countries, cold medicine and antipyretic analgesics can be easily bought from pharmacies. Patient awareness and the need to consider potential risks before taking OTC analgesics are increasing [24]. Recently, patients are refraining from going to the pharmacy to buy medicines to prevent infection, and it is now possible to purchase OTC online [25], potentially increasing the number of patients who take these medicines for prolonged periods [26]. Although the prevalence of these side effects is low with respect to drug-induced lung disease, drug sellers and patients must understand and pay attention to the risks associated with OTC cold medicines. Furthermore, in the differential diagnosis of interstitial pneumonia, we should consider drug-induced interstitial lung disease due to OTC cold medicine, which patients have been taking not only for weeks or months but also years.

## Conclusion

It should be noted that even OTC cold medicine, which generally have few side effects, might be addictive and that long-term administration might cause drug-induced interstitial lung disease. In this case, although acetaminophen was considered to be the causative drug, DLST was negative. Further development of a method for identifying the causative drug is desired.

## Data Availability

Not applicable.

## Abbreviations

OTC: Over-the-counter
DLST: Drug-induced lymphocyte stimulation test
